# Age-dependent histomorphometric evolution of the corpus spongiosum^1^

**DOI:** 10.1590/ACB351203

**Published:** 2021-01-20

**Authors:** Gino Pigatto, Marcelo Zeni, Eduardo Felippe Melchioretto, Gustavo Lenci Marques, Thiago Hota, Rogério de Fraga

**Affiliations:** I Fellow Master degree, Postgraduate Program in Surgical Clinic, Universidade Federal do Paraná, Curitiba-PR, Brazil.; II Msc, Department of Clinical Surgery, Health Sciences Sector, Universidade Federal do Paraná, Curitiba-PR, Brazil.; III Msc, Department of Clinical Surgery, Health Sciences Sector, Universidade Federal do Paraná, Curitiba-PR, Brazil.; IV PhD, Department of Clinical Surgery, Health Sciences Sector, Universidade Federal do Paraná, Curitiba-PR, Brazil.

**Keywords:** Aging, Elastic Tissue, Collagen, Urethra, Rats

## Abstract

**Purpose:**

To quantify the age-dependent changes in the relative volume of elastic fibers, collagen fibers and the proportion of collagen types I/III in the corpus spongiosum of rats.

**Methods:**

Forty-eight rats, raised under similar conditions, were divided into four groups (G1 to G4) and underwent penectomy at the ages of 6, 9, 12 and 24 months, respectively. Histological sections from the middle segment of the penis were stained with Weigert’s resorcin-fuchsin, Masson’s trichrome and Picrosirius red, the volumetric density of elastic fibers, collagen fibers and the proportion of collagen types I and III in the corpus spongiosum were determined by stereological analysis.

**Results:**

A reduction in the proportion of collagen I/III between the groups G3 and G4 (p < 0.048) was observed. In the volumetric analysis of elastic fiber, we observed a significant rise between the groups G2 and G3 (p < 0.03) and a reduction of the volume between the groups G3 and G4 (p < 0.01). However, there was no difference in the quantity of total collagen between the groups (p > 0.54).

**Conclusions:**

Aging in rats did not change the quantity of total collagen but reduced the proportion of collagen types I/III and the volume of elastic fibers.

## Introduction

The process of aging impacts the urinary system and affects the functioning of essentially all urinary organs. A number of these changes have a direct impact on genitourinary health, urologic function and clinical decision-making related to urologic care.

The changes associated with aging can particularly affect the functioning of the lower urinary tract and cause urinary incontinence and urinary irritative symptoms^1^^,^ 2, collectively termed as lower urinary tract symptoms (LUTS), which are frequently attributed to benign prostate enlargement. However, recent evidence suggests that this is a poor explanation for a complex problem, since LUTS can be characterized by structural and physiological changes in most parts of the inferior urinary tract, not just the prostate^3^. Notably, alterations in the quality and quantity of structural fibers in the extracellular matrix, such as collagen and elastic fibers, also occur. A good example of the clinical impact on the changes in the structural fibers of the corpus spongiosum (CS) is the increased urethral stiffness, resulting in impaired urethral flow without points of stricture after correcting hypospadia^4^. Therefore, it is reasonable to think that similar alterations in the extracellular matrix of the CS may occur with an increase in age as well.

With aging, the need for auxiliary procedures that tend to be frequently performed via endoluminal therapy also increases. However, these procedures often result in scarring of the CS that may be worsened with aging. Therefore, there is a need to understand the tissue composition and the natural physiology of the CS with aging in order to avoid surgical complications^5^^,^ 6.

It should also be noted that several changes in the female urethra associated with age have been well described; however, no such studies on the male urethra exist^7^. Therefore, understanding the structural changes due to the aging of the male urinary tract is crucial for comprehending the physiological changes in micturition and the etiology of the acquired symptoms and for expanding our knowledge about the ways that urethral scarring occurs in older men. Moreover, we found that very few studies describe the stereological changes in the CS. This study aimed to evaluate the age-dependent changes in the quantity of total collagen and elastic fibers and the proportion of immature and mature collagen forms in the corpus spongiosum (CS) of rats.

## Methods

This study followed the ethical principles of animal experimentation established by the Brazilian College of Animal Experimentation (COBEA) and the norms of the Canadian Council on Animal Care (1993). It was approved by the Ethics Committee on Animal Experimentation (CEEA), Department of Biological Sciences of the Universidade Federal do Paraná, as part of the project “Anatomical and physiological evaluation of male urogenital aging” process No. 23075.032620/2010-10.

Forty-eight male Wistar rats (*Rattus norvegicus* var. *albinus*), maintained under a light-dark cycle of 12 h and controlled temperature (22 °C) and receiving water and food [standard pellet diet; Nuvilab-Nuvital, Curitiba (PR), Brazil] ad libitum, until they were sacrificed. Rats were divided in four even groups, kept in even conditions until the age each group was ascribed for the time of the sacrifice for the study. The rats were randomly divided into four groups of 12 rats each (G1, G2, G3, and G4). The rats underwent sacrifice and resection of the penis and other organs for research at the ages of 6 (G1), 9 (G2), 12 (G3) and 24 (G4) months of age. For the sacrifice procedures, the rats were anesthetized intraperitoneally with a solution of ketamine hydrochloride (57.67 mg/mL) and 2% xylazine hydrochloride at a dose of 1 mL/kg body weight. A one-punch cardiac puncture was performed and cardiac arrest was induced by exsanguination. This procedure was followed by resection of the penis and kidneys, bladder, liver, brain, heart and aorta for use in multiple other researches of the aging process in a multidisciplinary.

### Histological processing

**Figure 1:**
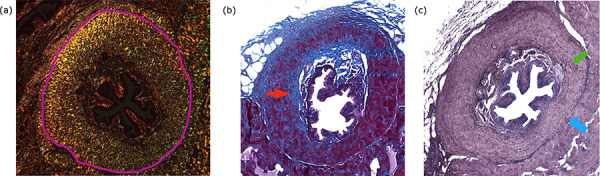
Corpus spongiosum stained with (a) Picrosirius red staining method visualized under polarized light. The fibers stained in the specter between red and yellow are collagen type I and those in green are collagen type III. The purple line delimitates the area of CS. **(b)** Corpus spongiosum stained with Masson’s trichrome staining method. Blue areas indicated by the red arrow are collagen fibers. **(c)** Corpus spongiosum stained with Weigert’s resorcin-fuchsin method. The blue arrow indicates the border of the corpus spongiosum and green arrow indicates elastic fibers.

The penis of each rat was laid on a horizontal surface and the middle third segment was resected, as previously described. After tissue fixation in 10% formalin, the segment was fixed for 16 h and later dehydrated using pure xylene and 70% alcohol. Finally, the sections were embedded in paraffin and 5 μm sections using a microtome (American Optical, Spencer AO 820) were prepared. Ten consecutive sections were mounted on slides and stained using Picrosirius red for distinguishing collagen type I and III, Masson’s trichrome for total collagen and Weigert’s resorcin-fuchsin method for visualizing elastic fibers. The images of the slides were captured using a video camera coupled with an optical microscope at 400 × magnification for the sections stained with Masson’s trichrome and Weigert’s resorcin-fuchsin methods. To capture the images of Picrosirius red staining, we used an optical microscope at 400 × magnification under polarized light to differentiate collagen type I (red fibers) from Type III (green fibers). We used the Zen 2.3 software (blue edition) from Carl Zeiss Microscopy GmbH, 2011 for scanning and editing the images (Fig. 1).

In order to perform the stereological analysis, we used the Image Pro Plus software, from Media Cybernetics, to calculate the total area of CS and the percentage of elastic fibers, total collagen fibers and collagen types I and III. This method was used to determine the area occupied by the spectrum of colors that represent a particular fiber in the CS. Such color area quantification was performed using HSI histogram-based color segmentation, after color equalization by the same software. The color area was divided by the complete area of CS to determine the percentage of CS occupied by that specific fiber. The areas of collagen types I and III were calculated in a separate manner and the area of collagen type I was divided by the total area of collagen type III to determine their ratios.

### Statistical analysis

A Pearson correlation statistical test was used to evaluate the association between the ages of rats and the relative areas of collagen and elastic fibers in the CS. Tukey’s multiple comparison test was used to assess the statistical significance of the differences in relative areas observed between the groups. A significance level of 95% was considered (p < 0.05) as statistically significant.

## Results

**Figure 2:**
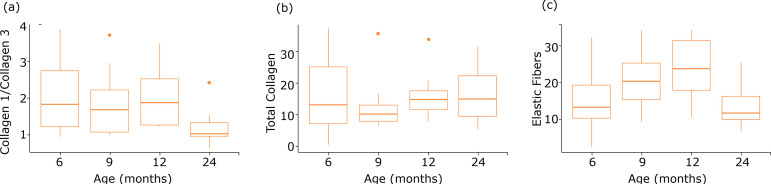
Graphs representing the correlation between months (ages) and (a) collagen type I/III, **(b)** total collagen and **(c)** elastic fibers.

The analysis of variance indicated significant difference between the proportions of collagen in at least one pair of groups. Tukey’s multiple comparison test identified significant differences between G3 and G4 with the p-value adjusted to 0.048. However, the other contrasts were not significant. The Pearson’s correlation coefficient estimated between the ages and the proportion of collagen types I/III was 0.4, with the p-value of 0.004, indicating that an increase in the age is associated with diminution in the proportion of collagen types I/III (Fig. 2). No significant differences between the total collagen of different age groups were found. Pearson’s correlation coefficient was estimated to be 0.09 with the p-value of 0.54 (Tabela 1).

**Table 1:** The proportion of collagen type I and III, distribution of total collagen and distribution of elastic fibers between the groups, represented by mean and standard deviation.

Group		Mean	Standard deviation
Proportion of collagen type I and III			
	G1	2.053	1.02
	G2	1.913	0.85
	G3	2.056	0.72
	G4	1.192	0.48
Distribution of total collagen			
	G1	15.83	12.68
	G2	12.42	8.09
	G3	15.96	7.06
	G4	16.82	9.23
Distribution of elastic fibers			
	G1	15.7	9.14
	G2	21.26	7.59
	G3	25.59	8.36
	G4	15	6.85

The analysis showed a significant difference between at least one pair of ages. Tukey’s multiple comparison test revealed a significant difference between G1 and G3, with the adjusted p-value of 0.03, and between G3 and G4, with the adjusted p-value of 0.01. However, the other contrasts were not significant. In this case, we observed an increase in the volume of elastic fibers until 12 months and then a significant decrease within 24 months, which is possible.

## Discussion

We observed the impact of the aging process in the extracellular matrix of the CS based on our results. These changes encompassed an initial increase followed by a decrease in the amount of elastic fibers and proportion of collagen type I/III.

In this study, we found an initial increase followed by a reduction in the quantity of elastic fibers with age. These changes, marked by a decrease in the volume of elastic fibers at the later stage, could be attributed to the late-onset of hypogonadism related to aging, as explained by Pradidarcheep^10^. Reforcing this theory, a similar change in the density of elastic fibers was found by Borges *et al.* 11, in a study comparing extracellular matrix morphology in neutered versus intact CS of cats. This study showed that neutered cats presents a smaller density of elastic fibers in the CS compared with intact cats.

da Silva *et al.* 12 also found elastic fiber changes in a study on human urethral plate in hypospadias repair associated with aging, where the number of elastic fibers reduced drastically with age. However, no initial increase in elastic fibers was observed. This is different finding from Gallo *et al.* 13 and Abidu-Figueiredo *et al.* 14, Gallo *et al.* 13 studied the modifications of erectile tissue components in the penis during the fetal period in Humans, and showed a significant increase of the elastic and collagen fibers during the early stages of development of CS. Abidu-Figueiredo *et al.* 14 studied rabbits penises from 30 to 730 days and found a progressive increase in the density of elastic fibers in the CS during the whole period. Since the tissue composition between the urethral plate of hypospadias and the healthy urethra differs, the heterogeneity between the samples of the Da Silva’s study and the others could justify these different results.

The elastic fibers in the extracellular matrix is known to affect the elasticity of the tissue and, therefore, may enhance obstructive symptoms, as demonstrated by Idzenga *et al.* 4. Idzenga *et al*.^4^ explained that after surgical repair the stiffness of the distal urethra limits the flow rate due to the inability to increase the diameter of the urethra under the same pre-determinate bladder pressure. In another study, Jinjin *et al.* 6 developed a model in which the stiffness of the fibrous prostate tissue was directly related to LUTS, regardless of the prostate size, corroborating the theory that increased CS stiffness contributes to reducing the flow rate.

By analyzing the decrease in the ratio of collagen type I/III, we found a similar result to Rodrigues *et al.* 16,who also studied the changes in the extracellular matrix of rats for a period of 44 weeks. In this study, he demonstrated that diabetes mellitus can worsen the negative impact of aging in collagenous tissue. The increased proportion of collagen type III represents increased fragility of the CS tissue, since these fibers are not as resistant to tension as collagen type I. Less resistance due to a decrease in the ratio of collagen type I/III, associated with less elasticity due to a decrease in the elastic fibers predisposes the old CS to rupture. Therefore, re-enforcing the use of very delicate and narrow surgical instruments when performing a surgery on the urethra in older adults could avoid lacerations, spongiofibrosis, and strictures^17^.

Our study did not find any significant change in the quantity of total collagen with the increase in age. This finding contradicted from the aforementioned study by da Silva *et al*.^12^ and Rodrigues *et al*.^16^ that showed an increase in the amount of total collagen with increasing age. However, this difference can be justified by the fact that our control group consisted of samples from the adult population, whereas the other two studies used a younger population as their control. Therefore, we can say that increase in the total collagen found in those studies might be due to the proper development of CS and not aging.

Additionally, our findings provide a better understanding of the age-related changes in the CS and thereby creates a base for developing new strategies to treat urinary system-related diseases and prevent the disease pathology. However, the greatest limitation of our study is that we cannot infer whether the exact same process occurs in humans as well and therefore more studies on human CS should be done. Further studies are also needed to correlate these histologic alterations with the direct impact on LUTS and the healing process in humans.

## Conclusion

Aging has a considerable impact on the composition of the extracellular matrix. These changes occur for elastic fibers as well as for collagen fibers.
